# A comprehensive survey of protein palmitoylation in late blood-stage *Plasmodium falciparum*

**DOI:** 10.1186/1475-2875-9-S2-P16

**Published:** 2010-10-20

**Authors:** Matthew L Jones, Mark O Collins, Jyoti S Choudhary, Julian C Rayner

**Affiliations:** 1 Sanger Institute Malaria Programme, Wellcome Trust Sanger Institute, Cambridge, Cambridgeshire CB10 1SA, UK

## 

Protein palmitoylation, the addition of a 16-Carbon saturated fatty acid to cysteine residues, is a common post-translation modification of both peripheral and integral membrane proteins. As a reversible modification, palmitoylation is essential for the dynamic regulation of protein function—controlling, in many cases, protein localisation both to and within specific membrane domains, protein stability, and protein activity. Using two complementary techniques, one relying on the exchange of palmitoyl groups with a biotin linker and the other relying on metabolic labelling of cells with a palmitic acid analogue, we have purified the total catalogue of palmitoylated protein from *Plasmodium falciparum* schizonts. The use of these two complementary techniques in tandem with SILAC based mass spectrometry to allow robust quantitative MS have allowed the identification of more than 250 palmitoylated proteins in late blood-stage *P. falciparum* parasites. This represents the first global analysis of protein palmitoylation in any *Plasmodium* parasite and has identified palmitoyl-proteins involved in protein secretion, protein export, drug resistance, signalling, development, and invasion. Furthermore, we have found that palmitoylation is essential for the completion of the blood-stage life cycle and that this modification directly regulates the stability of at least two proteins essential for invasion, PfGAP45 and PfMTIP.

